# Statistics of Fracture of the Cranium

**Published:** 1852-03

**Authors:** Frederick D. Lente


*Statistics of Fracture of Cranium.* By Frederick D. Lente, M. D.—Perhaps the reader, who has not had a large experience in fractures of the skull, will be astonished, upon a hasty examination of the results of the table, at the great mortality, 106 out of 128, and may recall to mind a number of such injuries, a majority of which recovered. But if he will examine more carefully into the *nature* and *effects* of the injury in the individual cases, he will perhaps be equally surprised at certain recoveries which did take place. The injuries are, indeed, almost all of an extreme character, admitting of but little hope of recovery, having been for the most part, occasioned by falls from a height upon the head, an incident, most likely of all to prove fatal, or by the fall of a heavy body from a height upon the head, producing not only extensive fracture of the skull, with depression, often extending into the base, but in many cases laceration of the membranes of the brain, and of the brain itself, and rendering recovery almost hopeless. But a brief abstract of the nature of the injury in each case, as far as our data will allow us to give it, may place the subject in its proper light, promising, that we have been guided by conjecture in no case, only stating as facts what was demonstrated fully by *ante* or *post-mortem* examination of the patient. Thus, in many cases, where the patient recovered, where no autopsy could be obtained, we have suspected, upon good grounds, fracture not only of the calvarium, but base, but have included no such case in the table.

Of the 128 cases, the exact extent of the fracture was ascertained in 95. In 45 of these the fracture involved both calvarium and base.

In 31 it involved the calvarium alone.

And in 20 the base alone.

In 26, or 20-31 per cent., both the brain and its membranes were lacerated; and in 4, the membranes alone. Of these 30 cases, only 3 recovered. In 10 cases, there was *hernia cerebri*, only 2 of which recovered. In 19 cases, or nearly 15 per cent., the fracture of the skull was complicated with other fractures, most of them very serious, and many of them almost sufficient of themselves to produce a fatal result.

*Fracture of the base of the Skull.*—In sixteen of the fractures involving the base of the skull alone, the manner of the accident is mentioned. In thirteen it occurred from a fall from a height upon the head. In two, from the fall of a heavy body from a height upon the head; and in one from being beaten over the head with a club. Almost all these fractures, it will be seen, occur from falls upon the head, the base of the skull being thus forcibly impelled against the spinal column, and thus broken, the fracture being doubtless due, in some measure, to *contre coup.*

*Separation of the Sutures.*—In *three* cases, there was separation of the sutures without any fracture. In *four*, separation of the sutures, accompanied by fracture. In all these cases, the anchylosis of the sutures, judging from the condition of those uninjured, was perfect. In *five* out of the seven cases, the injury was occasioned by a fall from a height, striking upon the head; in one from a blow with a club. This is a rare form of fracture, and only occurs, according to the authorities, from a very severe injury.

*Fracture of the internal table without injury of the external.*—In no case was this precise condition of things observed; but in two instances there was a very near approach to it, there being a mere fissure of the external plate, with fracture, attended by considerable depression of the internal. In one of them, the specimen of which is preserved in the pathological cabinet of the hospital, the fissure of the external table was so very slight as to be undiscoverable upon a first examination of the calvarium after its removal.

In two cases there was fracture of the calvarium by *contre coup.*

*Condition of the Pupils.*—In all the cases, twelve in number, where the extravasation or depression was confined to one side, and the pupils affected differently, the *dilatation* was on the *same side* with the cause of compression. In none on the opposite side. But, in many cases, where this cause was situated exclusively on one side, no particular difference was noticed in the condition of the pupils, they being entirely unaffected, or affected alike. In *twelve* cases, in which the symptoms of *compression* were well marked, the patients not being in immediate danger of death, the pupils were *dilated* in *ten*, (10,) contracted in *two* (2).

*Paralysis.*—In *eleven* cases in which there was hemiplegia, whether partial or total, rarely the latter, and where the injury was mostly confined to one side or the other, it was found to affect the opposite side in ten (10,) the same side in one.

*Trephining.*—In forty-five cases in which this operation was resorted to, not distinguishing between the application of the trephlne proper and of the various instruments appertaining to a trephining case, *eleven*, or about one-fourth, recovered.

The operation was performed *prophylactically* in ten (10,) of which three were cured; *therapeutically* in thirty-two (32,) of which eight were cured. In three of the latter, the operation was secondary, of which two recovered. In seven of the cases trephined, the fracture was simple. In five cases, incision of the *dura mater* was practised, of which none recovered.

In no case did death follow the receipt of the injury until after the lapse of some hours, even in the most desperate cases; nor does it appear to be possible for an ordinary blow upon the head, producing fracture of the skull, to cause immediate death. The nearest approach to it I ever saw, was just previous to the termination of my term of service in May last; I was called into the yard to see a man in a carriage, who, it was said, had just fallen from the rigging of a ship to the deck, striking upon his head; this was the history obtained from his two shipmates who brought him to the hospital. Upon examination, I found the case hopeless; the man was perfectly dead, and had given no signs of life after the accident; he was not removed from the carriage. I could detect no fracture of the skull, and none of the neck, and yet either might have existed; probably a half hour or hour elapsed between the receipt of the injury and his arrival at the hospital. In a recent criminal trial of great interest, it will be recollected that, at one stage of the proceedings, it was much discussed whether a blow upon the head with an ordinary weapon capable of inflicting death, could produce this result instantaneously; many eminent surgeons were examined, and the general impression was, that the thing was exceedingly improbable if not impossible, and the question was thus decided.—*Ibid.*

